# First confirmed case of nasal pythiosis in a horse in Thailand

**DOI:** 10.1099/jmmcr.0.005136

**Published:** 2018-01-09

**Authors:** Walaiporn Tonpitak, Watcharapol Pathomsakulwong, Chulabha Sornklien, Theerapong Krajaejun, Suppathat Wutthiwithayaphong

**Affiliations:** ^1^​ Department of Microbiology, Faculty of Veterinary Medicine, Mahanakorn University of Technology, Bangkok, Thailand; ^2^​ Equine Clinic, Faculty of Veterinary Medicine, Kasetsart University, Kamphaeng Saen campus, Nakhon Pathom, Thailand; ^3^​ Department of Pathology, Faculty of Medicine, Ramathibodi Hospital, Mahidol University, Bangkok, Thailand; ^4^​ Clinic for Horse, Faculty of Veterinary Medicine, Mahanakorn University of Technology, Bangkok, Thailand

**Keywords:** *Pythium insidiosum*, pythiosis, horse, Thailand

## Abstract

**Introduction:**

Pythiosis is caused by *Pythium insidiosum*, a fungus-like organism in the class *Oomycetes*. It can infect humans and a variety of animal species in tropical, subtropical and some temperate regions. Cases of animal pythiosis have occurred predominantly in horses in the skin and subcutaneous tissue at the limbs and in the ventral portion of thoracoabdominal wall - lesions in the nasal region are rarely reported. Moreover, although many human pythiosis cases have been reported in Thailand, no cases of animal pythiosis in Thailand have been reported.

**Case presentation:**

We report a case of pythiosis in a horse infected at the nasal cavity. Diagnosis was performed by zoospore formation by bait technique, immunohistochemical stain, immunochromatography and sequence analysis.

**Conclusion:**

The sequences of rDNA were 99 % and 96 to 99 % identical to GenBank isolates of *Pythium insidiosum* from two Thai human patients and horses from various countries, respectively. This represents the first confirmed report of nasal equine pythiosis in Thailand.

## Introduction

Pythiosis is caused by *Pythium insidiosum*, a fungus-like organism in the class *Oomycetes. P. insidiosum* mainly inhabits water and moist soil, utilizing aquatic plant blades to adhere and form motile biflagellate zoospores in its infective stage. The route of pythiosis infection is zoospore contact, especially with damaged skin, therefore the site of lesions is often a site of zoospore contact [1].

*P. insidiosum* can infect humans and a variety of animal species in tropical, subtropical and some temperate regions. Many human pythiosis cases have been reported in Thailand, but no animal pythiosis cases have been reported in this country [2, 3]. Cases of animal pythiosis have occurred worldwide, and the largest number of reported cases have been in horses. Infections in other species such as dogs, cats, cattle, sheep, birds and captive animals have also been reported [1].

Pythiosis lesions in humans are predominately in vascular and ocular forms. In animals, cutaneous forms are common in a variety of species. In dogs, however, the gastrointestinal form is most common [4, 5].

Equine pythiosis lesions are most commonly located at the skin and subcutaneous tissue at the limbs and in the ventral portion of thoracoabdominal wall - lesions in the nasal region are rarely reported. The lesions usually consist of a granulomatous nodule with ulcerative tissue or a fistula tract which contain kunkers, which are coral-like necrotic tissue. This is the typical pythiosis lesion in horses [6].

Pythiosis diagnosis is based on culture identification, antibody identification by ELISA, Western blot, immunodiffusion, immunochromatographic test and internal transcribed spacer (ITS) sequence analysis. Serodiagnostic analysis by immunochromatographic testing has been found to be rapid and its specificity, sensitivity and accuracy are 100 % [7].

This report describes a case of equine nasal pythiosis diagnosed by culture identification, sporulation baiting technique, immunochromatographic testing and ITS sequence analysis. It represents the first confirmed report of nasal equine pythiosis in Thailand.

## Case report

A four-year-old, Thai breed, seven-month-pregnant mare with a one-month history of a nodular mass at the right nasal septum was admitted to the Large Animal Teaching Hospital, Mahanakorn University in October 2015. The horse farm was located in Chonburi, central Thailand. The water for the horse was supplied from the reservoir next to the farm.

**Fig. 1.**
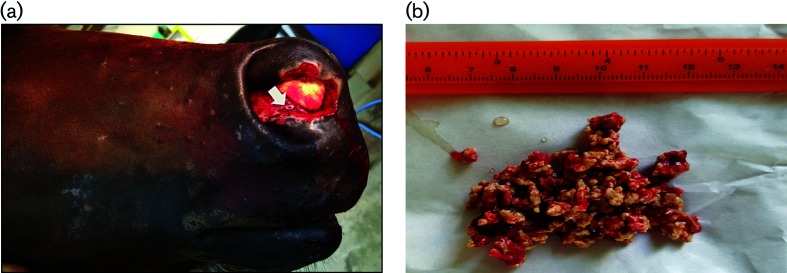
Clinical finding of infected horse. (a) Nasal mass lesion with ulcerative wound. (b) Coral-like necrotic tissues (kunkers) from the nasal mass.

The mass was approximately 10 cm from the muzzle; the size was approximately 3 cm in diameter with an ulcerative wound and serosanguineous discharge (Fig. 1a). The horse had massive muzzle pain, whereas all vital signs were normal. The presumptive diagnosis was an abscess. Two weeks after admission, many coral-like necrotic tissues were drained from the ulcerative nodule (Fig. 1b).

**Fig. 2.**
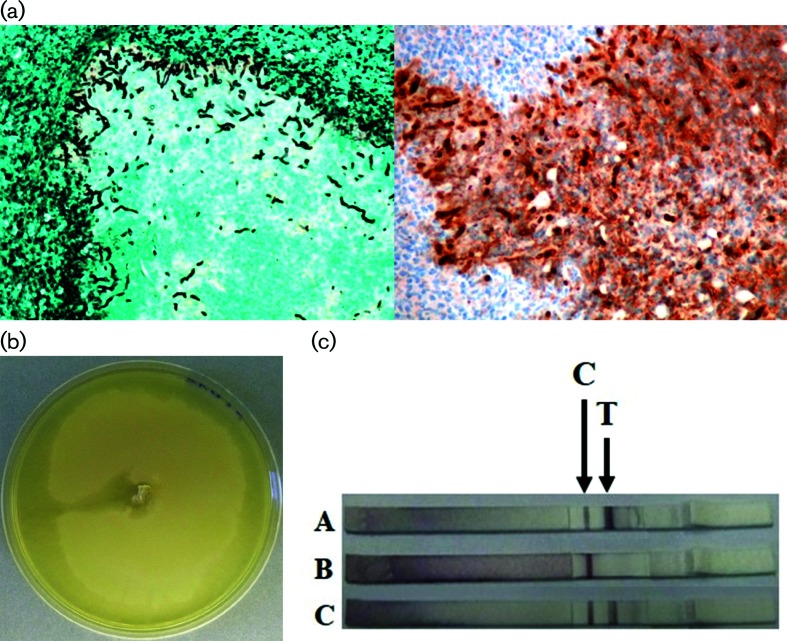
Diagnostic methods used for pythiosis confirmation. (a) Histopathologic demonstration of tissue biopsy: left, Gomori’s methenamine silver stain showing numerous sparse septate hyphae; right, immunostaining of the biopsy tissue with specific antibody to *P. insidiosum* showing positive staining and a large number of branching hyphae. (b) Culture of *P. insidiosum* isolated from kunkers on Sabouraud dextrose agar supplemented with chloramphenicol for 15 days. (c) Detection of IgG against *P. insidiosum* by immunochromatographic test using *P. insidiosum* antigen; A, positive control; B, negative control; C, horse serum sample; C, control line; T, test line.

Tissue from the biopsy were fixed in 10 % neutral buffered formalin and processed routinely for histopathology. Staining with Gomori’s methenamine silver stain revealed sparse septate hyphae similar to *P. insidiosum*. Immunohistological staining of the biopsy tissue with specific antibody to *P. insidiosum* tested positive and showed a large number of branching hyphae (Fig. 2a) [8].

Fungal cultures from fresh kunkers plated on Sabouraud dextrose agar supplemented with 0.05 mg ml^−1^ chloramphenicol were incubated in a 37 °C aerobic condition for 15 days and presented a submerged hyaline colony (Fig. 2b). The isolate was designated as Pi53. Zoospore production from the suspected *P. insidiosum* colony by bait technique was then performed [9]. Tubular zoosporangia on a grass blade were observed. Zoospores in sporangia were produced and released after incubation for 24 h.

A serum sample was collected for the detection of anti- *P. insidiosum* antibodies by a protein A/G-based immunochromatographic test [10]. Serum was diluted 1 : 5000 and tested with an immunochromatographic test strip - all tests were read with the naked eye within 30 min. Pool positive serum and negative serum from human patients were used as positive and negative control, respectively. A test result was considered positive if the test line and control lines appeared, while a test result was considered negative when only the control line was observed. The sample showed positive results like a positive control from a human patient pythiosis serum (Fig. 2c).

Fungal DNA extraction was performed using a salt-extraction protocol [11]. The ITS region of the rDNA sequence was amplified using the fungal universal ITS1 (5′-TCCGTAGGTGAACCTGAGG-3′) and ITS4 (5′-TCCTCCGCTAATTGATATGC-3′) primers [12]. The amplicons were sequenced using the ITSI and ITS4 primers, an ABI PRISM BigDye terminator cycle sequencing ready kit (Applied Biosystems) and an ABI 3100 Genetic analyzer (Applied Biosystems). The ITS sequence was deposited to the GenBank database under accession number LC199889 and analysed by blast (http://www.ncbi.nlm.nih.gov/BLAST). The result revealed a 97 to 99 % genetic identity to the sequence of *P. insidiosum* isolated from different hosts and geographic locations, which were available in the GenBank database. Furthermore the phylogeny analysis based on ITS sequences using the maximum-likelihood (ML) algorithm with 500 bootstrap replicates showed that the isolate Pi53 is most closely related to strains Pi14 (GenBank accession number LC199877) and Pi31 (GenBank accession number AB898120) isolated from Thai human samples whose phylogenetic tree is shown in another report [13]. The sequence was also subjected to blast analysis against isolates from horses in various locations, and showed identity to isolates classified in clade-II from horses in Japan, Papua New Guinea and Australia at 99 %, and isolates classified in clade-I from horses in Costa Rica and Brazil at 96 to 98 %.

Based on the diagnosis results, the horse was tranquillized with butorphanol 0.02 mg kg^−1^ and detomidine 0.01 mg kg^−1^ and then anaesthetized locally by 2 % lidocaine hydrochloride. Incision and drainage was carried out to remove granulation tissues and kunkers. A solution containing 50 mg amphotericin B, 6 ml DMSO and 44 ml lactate Ringer’s solution was infiltrated around the lesion and the fistula was filled with the remaining solution by using a setonization technique [14]. The wound was cleaned every day with diluted chlorhexidine, and a topical application with 2 % sertaconazole cream and cephalexine 30 mg kg^−1^ was given every 12 h for 10 days. Three weeks after surgery, the lesion had healed and the horse was discharged from the hospital.

## Discussion

*P. insidiosum* causes pythiosis in humans and animals. The site of the lesion is usually related to the site of the zoospore exposure. In horses, pythiosis commonly infects the limbs and ventral portion of the thoracoabdominal region. However, this case report demonstrates that the nasal cavity can be also affected in horses - as was reported in a previous report on horses and sheep in Brazil [6, 15, 16]. The route of zoospore contact in this case was probably drinking water supplied by the water reservoir near the farm. Pregnancy could have increased this horses’ sensitivity to infection due to a weakening of the immune system that can occur during this state - so the risk of zoospores contact from the environment in general is also possible. However, in our opinion, the water reservoir supplying the drinking water for the farm is the most likely source of infection.

The first attempted fungal culture from the biopsy tissue was not successful. Two weeks later, kunkers were observed and submitted to a diagnostic laboratory to isolate *P. insidiosum*. A successful *P. insidiosum* culture from these fresh kunkers revealed that fungal culture from fresh kunkers was more successful than that derived from tissue taken from granuloma lesions, as was also found in the study of Grooters and coworkers [17].

The most common cases of pythiosis reported in Thailand are human [2]. Until now, however, no cases of animal pythiosis in Thailand have been reported - although there have been sporadic unconfirmed cases of equine pythiosis. This may be due to a lack of efficient diagnostic methods. Although kunkers lesions in horses are typical and helpful for preliminary diagnosis, a definitive diagnosis should be performed. We used the immunochromatographic test and rDNA sequence analysis to assist in a definitive diagnosis. The immunochromatographic test using protein A/G-based immunochromatographic test strips was helpful for diagnosis because the result can be obtained within 30 min. The rDNA sequence analysis of the *P. insidiosum* isolate showed a close identity to two isolates from Thai human patients, which were classified in clade-II *P. insidiosum* [2, 13]. These represent the closest related isolates at 99 % sequence identity.

Definitive diagnosis based on immunohistochemical staining, immunochromatographic testing and rDNA sequence analysis confirmed that nasal granuloma of the horse in this report were infected by *P. insidiosum*. The present case report is the first confirmed diagnosis of pythiosis in a horse found in Thailand.

The clinical findings in this case were similar to cases of pythiosis reported at other sites of infection in horses - with kunkers being the typical lesion released from the granuloma mass. These findings, along with our report’s confirmation of the possibility of nasal infections, could be helpful for the clinical diagnosis of pythiosis in horses [18].
